# Supplementary material for: USP5-Beclin 1 axis overrides p53-dependent senescence and drives Kras-induced tumorigenicity
Source: Nat Commun. 2022 Dec 17;13:7799. doi: 10.1038/s41467-022-35557-y (PMC9759531; doi:10.1038/s41467-022-35557-y)
Supplement: Supplementary file 1 — Supplementary Information [file 41467_2022_35557_MOESM1_ESM.pdf]

Supplementary Figure S1

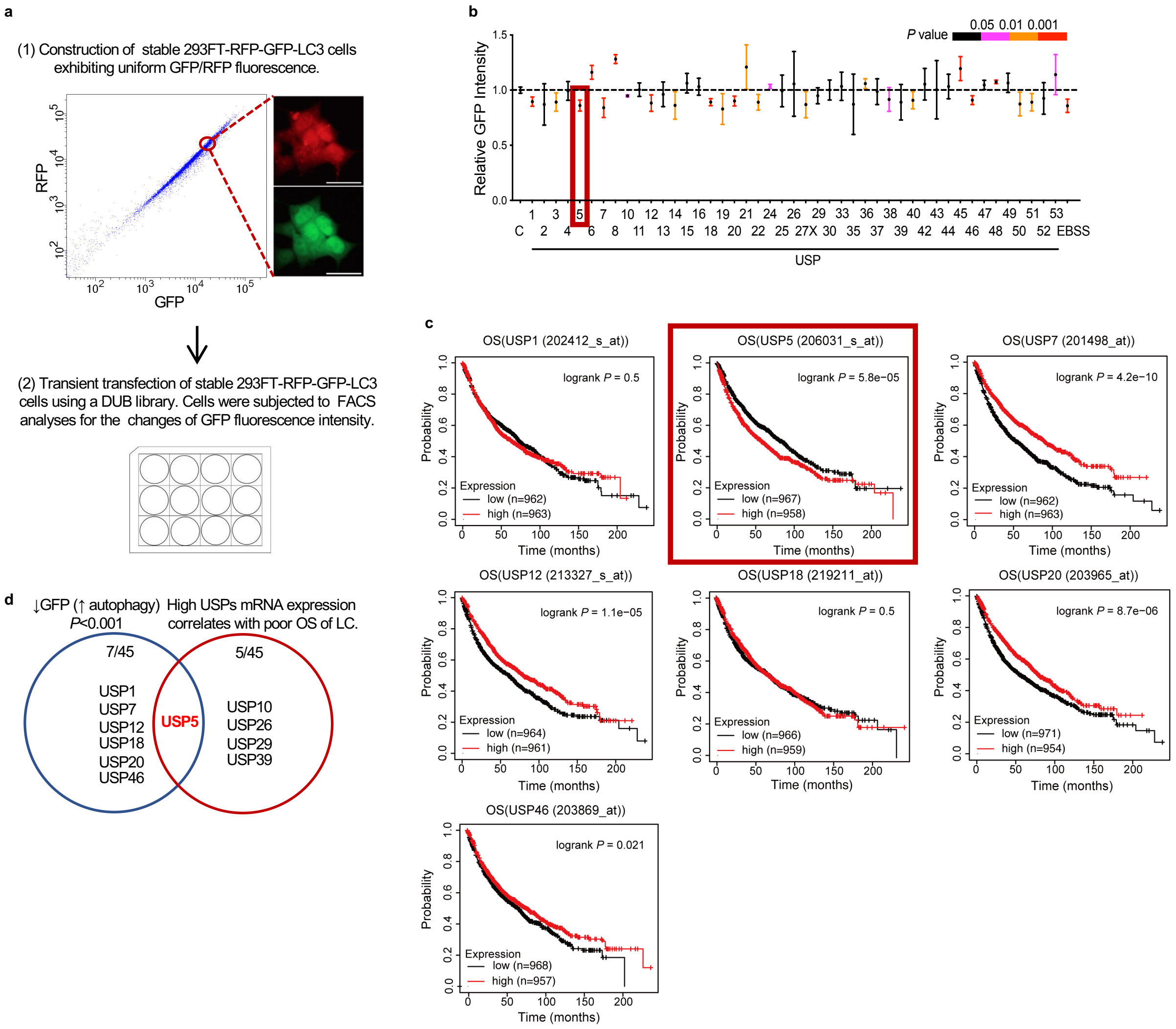

Supplementary Figure S1. High levels of USP5 facilitate autophagy and correlate with poor prognosis in lung cancer patients.

a. Construction of a reporter cell line used to screen genes encoding ubiquitin-specific proteases (USPs) involved in autophagy. (1) Stable 293FT-RFP-GFP-LC3 cells were established using pLenti-CMV-RFP-GFP-LC3 recombinant lentivirus[73]. Infected cells exhibiting uniform GFP/RFP fluorescence intensity were sorted and used in the study. (2) Expressing plasmids (pCMV6Entry) included in the DUB library consisting of 45 different USP genes (USP1-USP53) were transiently transfected individually into the stable 293FT-RFP-GFP-LC3 cells. Seventy-two hours post-transfection, cells were subjected to FACS analyses for the changes in GFP fluorescence intensity. The gating strategy was given in Supplementary Figure **S7a**. Reduced GFP intensity represents an increase in autophagy. Experiments were performed three times independently. The EBSS-treated cells were analyzed in parallel as a positive control to reflect increased autophagy. Scale bar=50 μm. **b**. The GFP fluorescent intensity was normalized to vector control. The normalized GFP intensity below 1.0 indicated the elevation in autophagy. Experiments were performed at least three times independently. Data were presented as mean ± SD. Comparisons were performed with two-tailed Student's t test. Exact *P* values were calculated accordingly and shown in Supplementary table **S3**. **c**. Overall survival (OS) plots generated from Kaplan-Meier Plotter database for mRNA expression of autophagy-inducing USPs as indicated in lung cancer patients. **d**. The Venn diagrams show that USP5 induced autophagy (decreased GFP fluorescence intensity) with *P*<0.001 and the expression of USP5 negatively correlates with poor prognosis in lung cancer patients.

Supplementary Figure S2

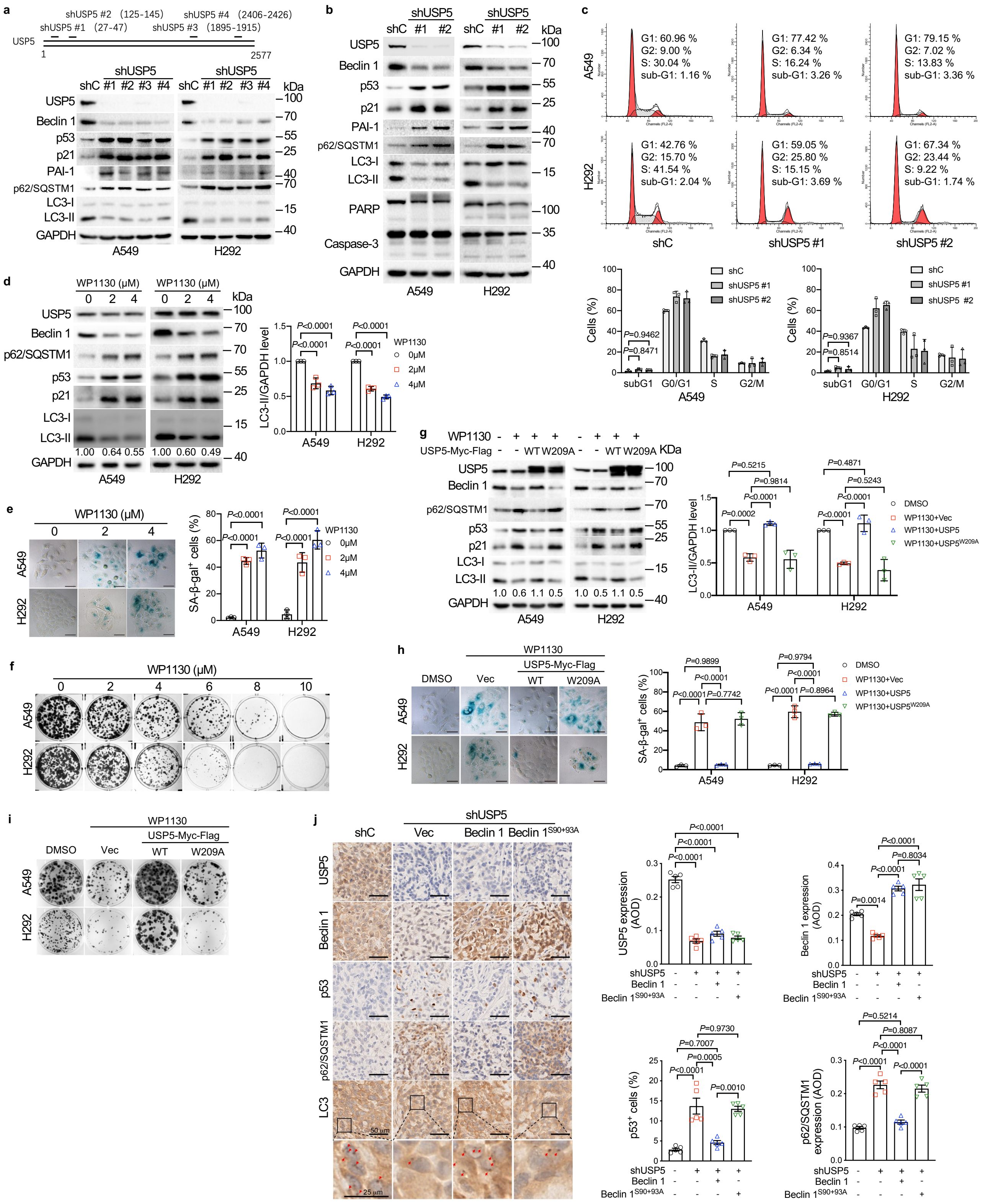

Supplementary Figure S2. Ablation of *USP5* or pharmacological USP5 inhibition inhibits Beclin 1-dependent cancer cell growth and induces cellular senescence.

a. A549 and H292 cells stably expressing shRNA targeting different regions of *USP5* (#1, #2, #3, #4) or control shRNA (shC) were subjected to western blot analyses. **b-c.** A549 and H292 cells stably expressing shRNA specific for *USP5* (#1 or #2) or control shRNA (shC) were subjected to western blot analyses and FACS analyses. The gating strategy was given in Supplementary Figure **S7b**. **d-f.** A549 or H292 cells were treated with the USP5 inhibitor WP1130 and were then subjected to western blot analyses (**d**), SA-β-gal staining (**e**) or colony formation assays (**f**). Quantification of LC3-II/GAPDH ratio was shown. Representative photos and quantification of SA-β-gal staining positive cells were presented. **g-i.** A549 and H292 cells stably expressing WT USP5 or $USP5^{W209A}$ treated with WP1130 were subjected to western blot analyses (**g**), SA-β-gal staining (**h**) or colony formation assays (**i**). Quantification of LC3-II/GAPDH ratio was shown. Representative photos and quantification of SA-β-gal staining positive cells were presented. **j**. The xenograft tumors from each group (n=5/group) derived from **Figure 1j** were subjected to IHC assays, using specific antibodies as indicated. Representative images were shown. Quantifications (Average Optic Density, AOD) of USP5, Beclin 1, p53, p62/SQSTM1 or LC3 were performed. Three independent experiments were performed (**a**-**i**). Data were presented as mean ± SD (**c**-**e**, **g**-**h**) or ± SEM (**j**). Comparisons were performed with two-way (**c**-**e**, **g**-**h**, **j**) ANOVA with Tukey's test. Scale bar=50 μm.

Supplementary Figure S3

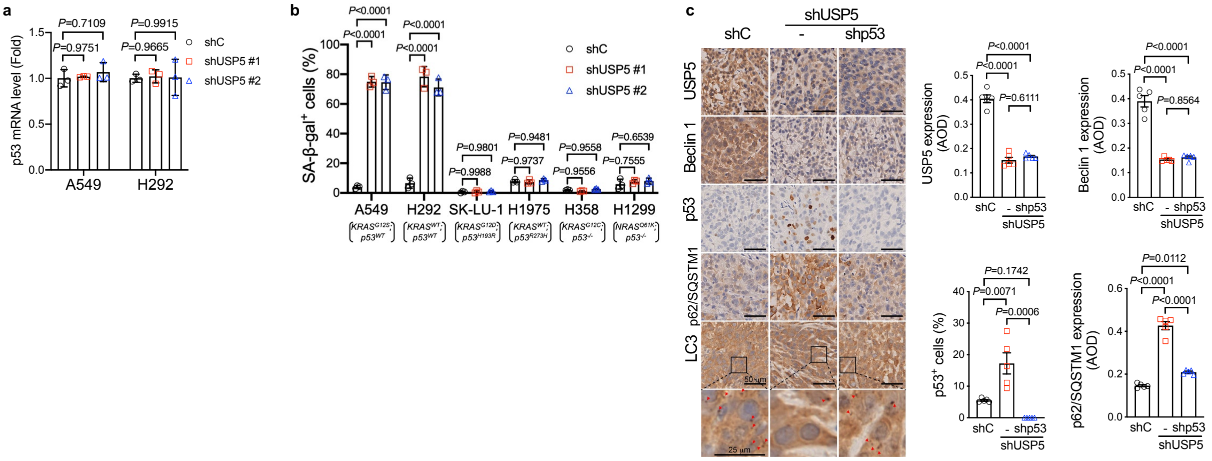

Supplementary Figure S3. Ablation of *USP5* leads to alteration of protein expression of Beclin 1, p53, p62/SQSTM1 and LC3, resulting in p53-dependent cellular senescence and suppression of xenograft tumor growth.

a. Knockdown of *USP5* does not significantly alter steady-state p53 mRNA levels. A549 or H292 cells stably expressing shUSP5 were subjected to qPCR analyses for p53 mRNA levels. Data were derived from three independent experiments and were presented as mean ± SD. **b. Knockdown of USP5 does not significantly alter SA-β-gal activity in lung cells harboring a mutant *p53* allele or *p53* null cells.** Indicated cells stably expressing shUSP5 were subjected to SA-β-gal staining. Data were derived from three independent experiments and were presented as mean ± SD. **c. Knockdown of *p53* restores cell proliferation suppressed by ablation of *USP5 in vivo*.** The xenograft tumors derived from **Figure 3f** (n=5/group) were subjected to IHC assays, using a specific antibody as indicated. Representative images were shown. Quantifications (AOD) of USP5, Beclin 1, p53, p62 or LC3 were performed. Data were presented as mean ± SEM. Comparisons were performed with two-way (**a**-**c**) ANOVA with Tukey's test. Scale bar=50 µm.

Supplementary Figure S4

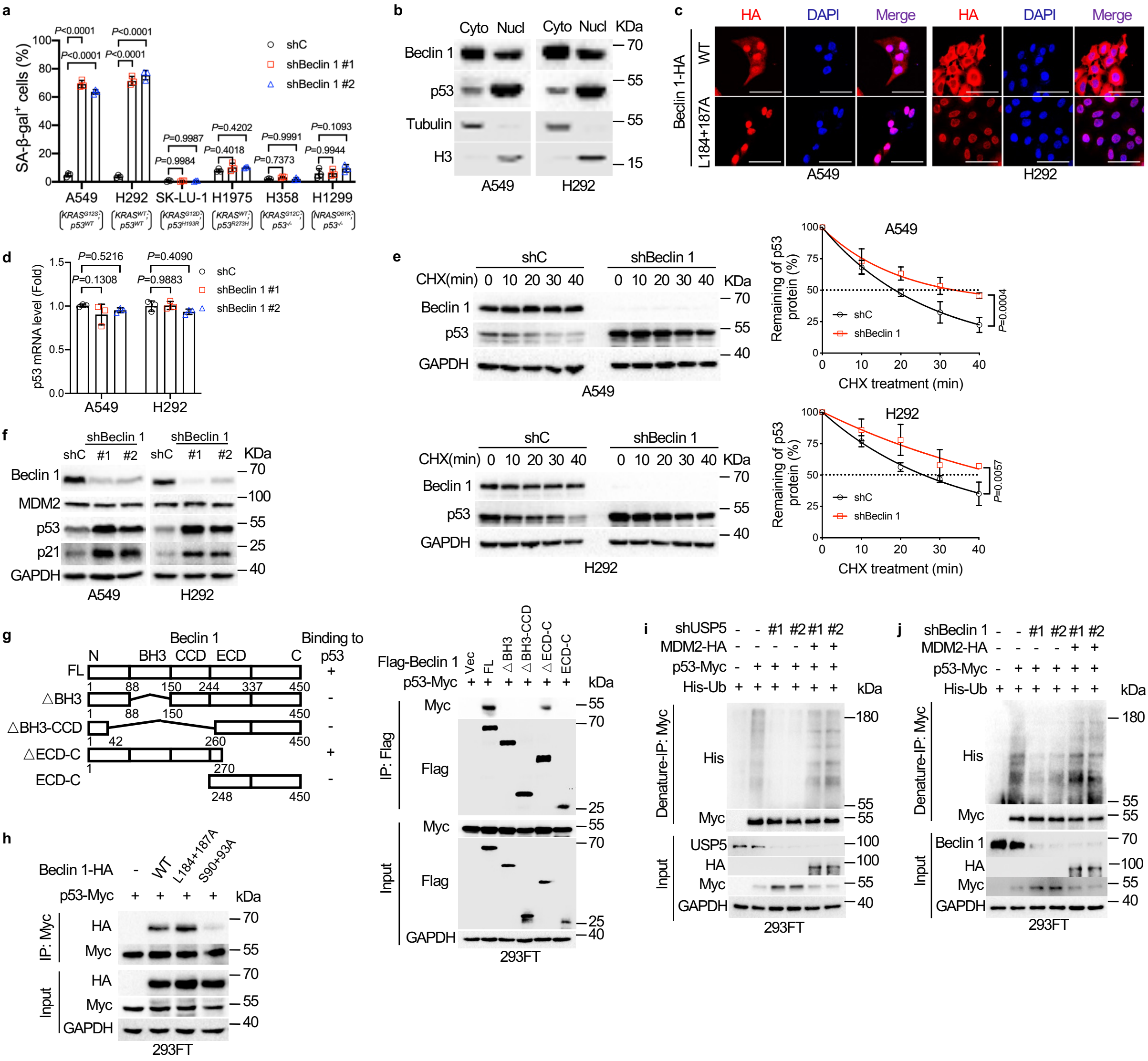

Supplementary Figure S4. Inhibition of *BECN1* stabilizes p53 to inhibit cell growth.

a. A549, H292, SK-LU-1, H1975, H358 and H1299 cells stably expressing shBeclin 1 (#1 or #2) were subjected to SA-β-gal staining. **b**. Total cell lysates derived from A549 or H292 cells were subjected to nuclear-cytoplasmic fractionation assays. **c**. A549 and H292 cells stably expressing Beclin 1-HA or Beclin $1^{L184+187A}$-HA were subjected to IF staining for Beclin 1 with HA antibody (red) or nuclei with DAPI (blue). Scale bar=50 μm. **d**. A549 or H292 cells stably expressing shBeclin 1 were subjected to qPCR analyses for *p53* mRNA levels. **e**. A549 or H292 cells stably expressing shBeclin 1 were subjected to p53 protein half-life assay. **f**. A549 or H292 cells stably expressing shBeclin 1 were subjected to western blot analyses for p53, MDM2 or p21. **g.** A schematic diagram of Beclin 1-p53 interaction (left panel). 293FT cells were transfected with p53-Myc and Flag-Beclin 1 (FL) or an indicated mutant construct. Total cell lysates were subjected to IP-western blot analyses (right panel). **h**. 293FT cells were transfected with p53-Myc and Beclin 1-HA or Beclin $1^{L184+187A}$-HA or Beclin $1^{S90+93A}$-HA. Total cell lysates were subjected to IP-western blot analyses. **i-j**. To examine the effects of MDM2 on USP5/Beclin 1 ablation-induced ubiquitination of p53, 293FT cells were co-transfected with indicated expressing plasmids. Cells were treated with 20 μM MG132 for 6 hours before collection. Ubiquitination of p53 was examined by denature-IP-western analyses. Three independent experiments were performed (**a**-**j**). Data were presented as mean ± SD (**a**, **d**, **e**). Comparisons were performed with two-way ANOVA with Tukey's test (**a**, **d**) or Bonferroni's test (**e**).

Supplementary Figure S5

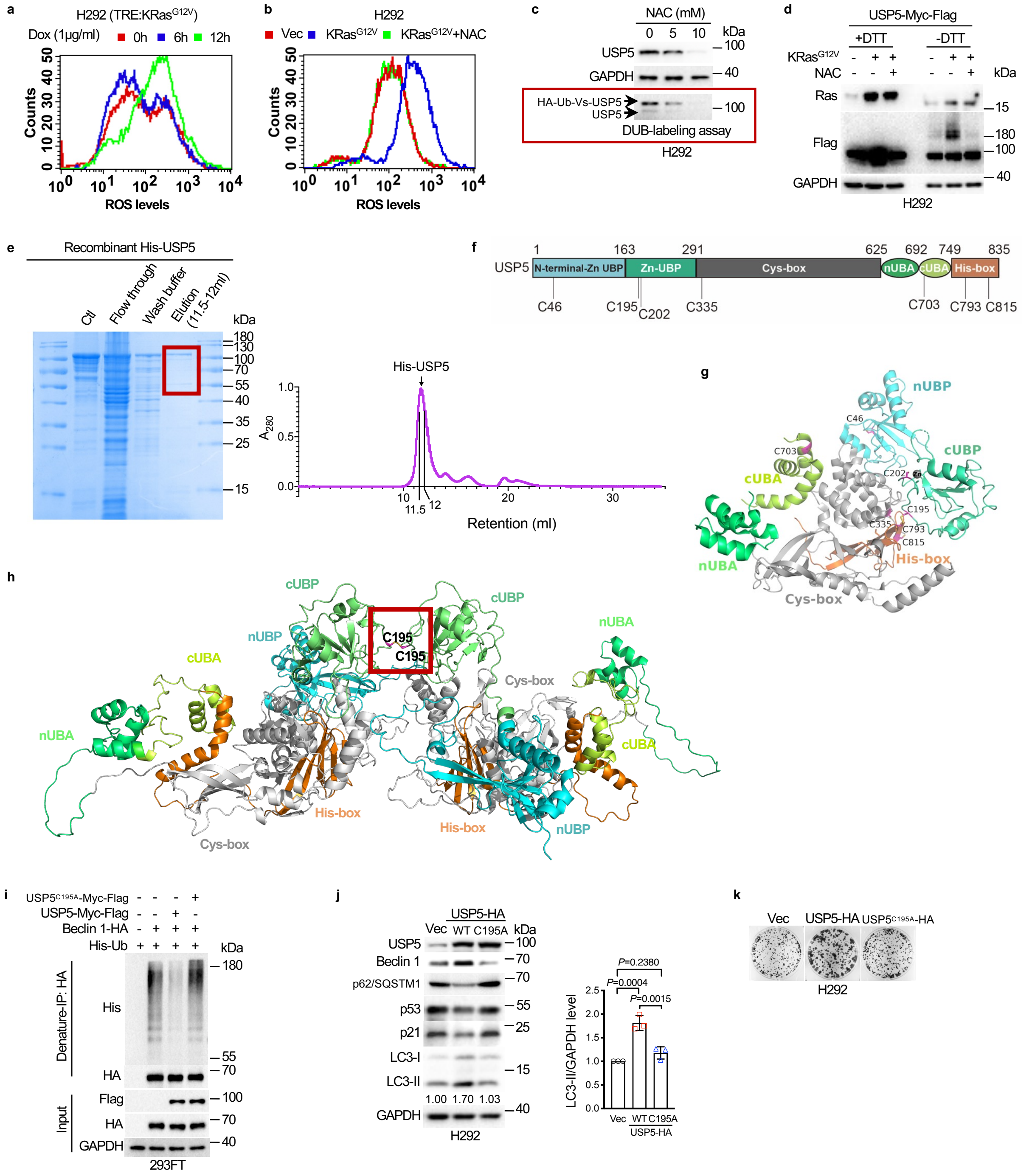

Supplementary Figure S5. ***$KRAS^{G12V}$*** **promotes USP5 dimerization in a ROS-dependent manner.**

a. H292 cells harboring doxycycline-inducible $KRas^{G12V}$ were treated with 1 μg/μL doxycycline at the indicated time intervals prior to FACS analyses for DCFA fluorescence. The gating strategy was given in Supplementary Figure **S7c**. **b.** H292 cells stably expressing $KRas^{G12V}$ were treated with 5 mM NAC for 24 hours prior to FACS analyses for DCFA fluorescence. The gating strategy was given in Supplementary Figure **S7c**. **c.** H292 cells were treated with 5 mM NAC for 24 hours, and then subjected to western blot analyses and the DUB-labeling assay for USP5 deubiquitination activity. **d.** H292-$KRas^{G12V}$/USP5-Myc-Flag cells were treated with 5 mM NAC for 24 hours prior to SDS-PAGE with or without DTT in the loading buffer, followed by western blot analyses. **e.** Recombinant His-USP5 protein generated in *E. coli* was subjected to the purification of the Ni^{+} column followed by SDS-PAGE and Coomassie Brilliant Blue (CBB) staining. **f.** A schematic diagram depicts USP5 protein structural domains. **g**. A partial USP5 protein crystal structure (3IHP [10.2210/pdb3ihp/pdb]) from the PDB database and the projected cysteines on the surface were shown. **h.** Full-length USP5 protein structures were predicted by *Alphafold2*[46] and the USP5 dimerization was projected by the *Gromacs*[47], in which a disulfide bond is formed between C195 residues of two monomers. **i.** 293FT cells co-transfected with expressing plasmids of Flag-Beclin 1, His-Ub, USP5-HA or $USP5^{C195A}$-HA were treated with 20 μM MG132 for 6 hours before collection. Ubiquitination of Beclin 1 was examined by denature-IP-western analyses. **j-k.** H292-USP5-HA or H292-$USP5^{C195A}$-HA stable cells were subjected to western blot analyses (**j**) or colony formation assay (**k**). Quantification of LC3-II/GAPDH ratio was shown. Three independent experiments were performed (**a**-**e,** **i**-**k**). Data were presented as mean ± SD and comparisons were performed with one-way ANOVA with Tukey's test (**j**).

Supplementary Figure S6

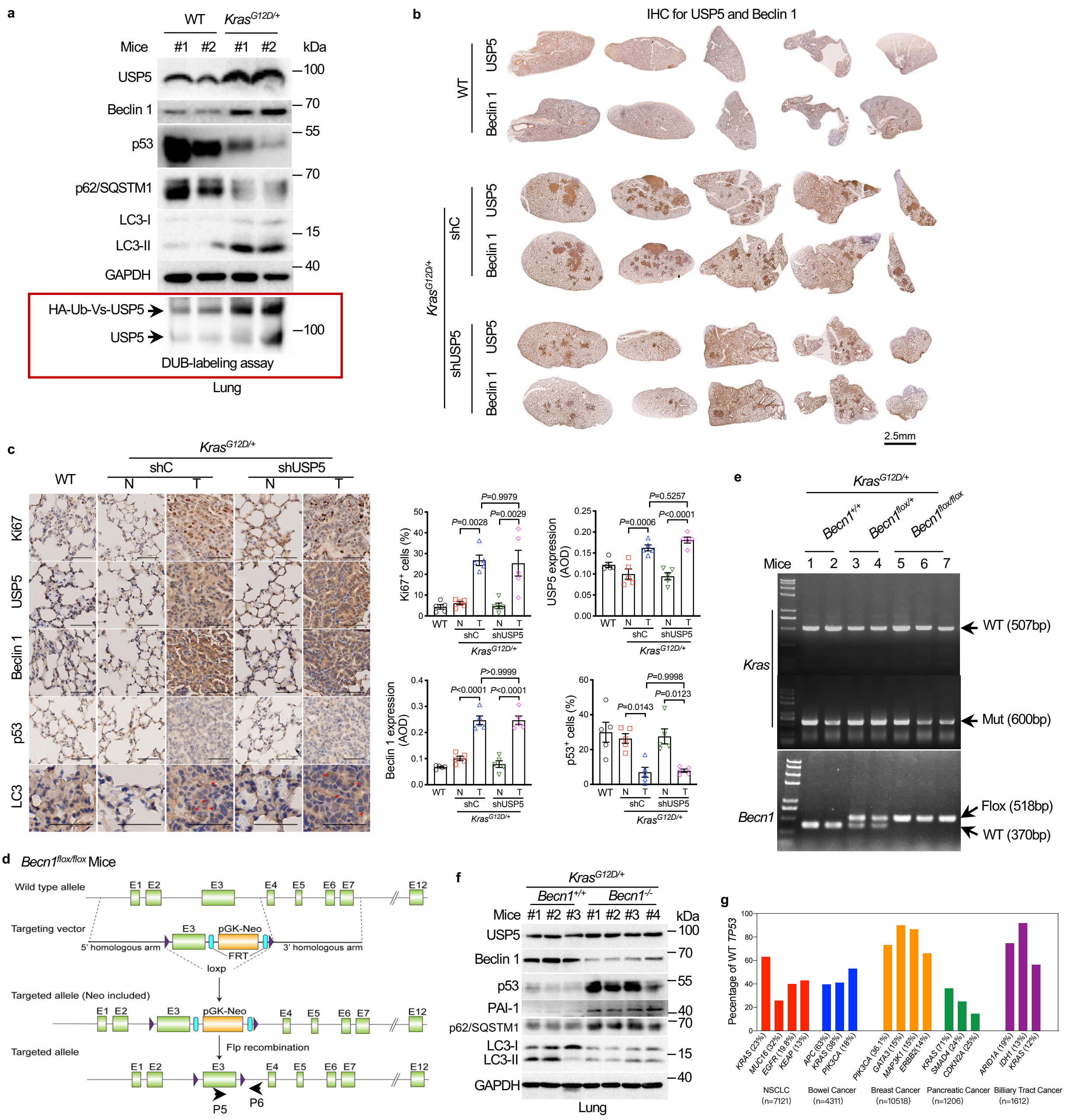

Supplementary Figure S6. Inhibition of *USP5* and *Becn1* suppress *Kras*-driven lung tumor growth.

a. Lung tissues derived from WT or *Kras*$^{G12D/+}$ mice were subjected to both western blot analyses and DUB-labeling assay after sacrifice. **b**. Lung lobes derived from Lenti-shC- or Lenti-shUSP5-treated *Kras*$^{G12D/+}$ mice were subjected to IHC staining and representative images of USP5 were shown (n=5). Scale bar=2.5 mm. **c**. Lung tissues derived from Lenti-shC- or Lenti-shUSP5-treated *Kras*$^{G12D/+}$ mice were subjected to IHC staining (n=5/group). Representative IHC images were shown and the corresponding quantification of USP5, Beclin 1, LC3, Ki67, or p53 was presented as mean ± SEM. Comparisons were performed with two-way ANOVA with Tukey's test. Scale bar=50 μm. **d.** Schematic illustrating the construction of the *Becn1*$^{flox/flox}$ mice. **e.** Genotyping of *Kras*$^{LSL\text{-}G12D/+}$;*Becn1*$^{flox/flox}$ compound mice by PCR. **f.** Lung tissues derived from *Kras*$^{G12D/+}$;*Becn1*$^{+/+}$ or *Kras*$^{G12D/+}$;*Becn1*$^{-/-}$ mice were subjected to western blot analyses after sacrifice. **g.** The incidence of wild-type p53 occurred in cancer patients with frequently mutated driver oncogenes. Data were analyzed by the cBioPortal database (https://www.cbioportal.org/). n = number of samples. Experiments were performed at least two times independently (**a**, **e**, **f**).

Supplementary Figure S7

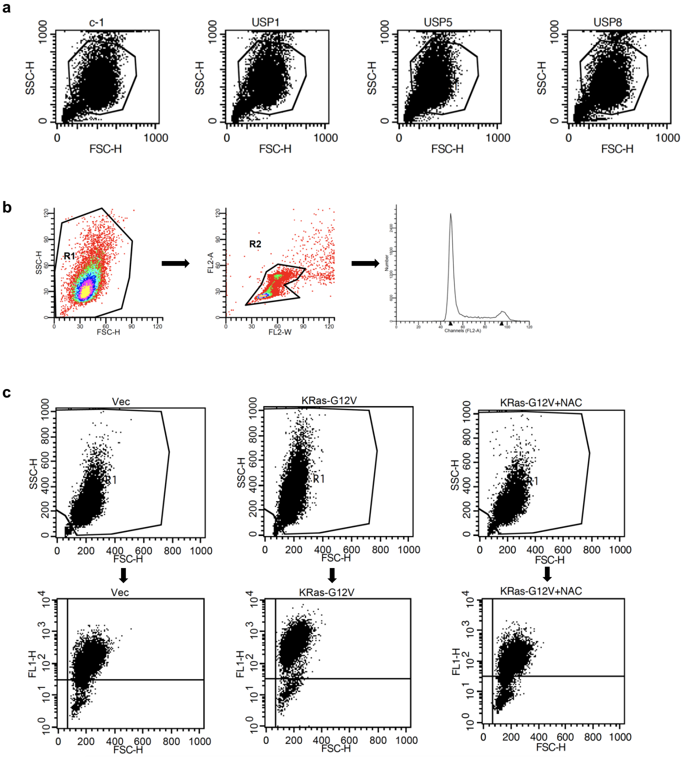

Supplement Figure S7. FACS gating strategy.

a. The representative FACS gating strategies for analyzing GFP fluorescent intensity were shown.

b. The representative FACS gating strategies for analyzing cell cycle were shown.

c. The representative FACS gating strategies for analyzing DCFA fluorescence were shown.

Supplementary Table S1. Primers were used in this study.

Target	Application	Sequence
Scramble	shRNA	TCCTAAGGTTAAGTCGCCCTCG
USP5	shRNA #1	GCTGTCAGTATTACCGACGAT
USP5	shRNA #2	GCCTCTACATCTGTATGAACA
USP5	shRNA #3	GTTTCTATGGCAACGAAGACG
USP5	shRNA #4	GCTCTTTGCCTTCATTAGTCA
Beclin 1	shRNA #1	CCCGTGGAATGGAATGAGATT
Beclin 1	shRNA #2	CTCAGGAGAGGAGCCATTT
p53	shRNA #1	GAGGGATGTTTGGGAGATGTA
p53	shRNA #2	CACCATCCACTACAACTACAT
BECN1 F	qPCR	AGGTTGAGAAAGGCGAGACA
BECN1 R	qPCR	GCTTTTGTCCACTGCTCCTC
P53 F	qPCR	CCTCACCATCATCACACTGG
P53 R	qPCR	GCTCTCGGAACATCTCGAAG
GAPDH F	qPCR	CCCGTGGAATGGAATGAGATT
GAPDH R	qPCR	CTCAGGAGAGGAGCCATTT
Primer 1 for WT *kras*	PCR	GTCGACAAGCTCATGCGGG
Primer 2 for WT and Mut *kras*	PCR	CGCAGACTGTAGAGCAGCG
Primer 3 for Mut *kras*	PCR	CCATGGCTTGAGTAAGTC TGC
P5 for *Becn1*	PCR	CGATGTTGCCCTCCTGTAAT
P6 for *Becn1*	PCR	TTGGATCTAGGAGCTTGCAT

Supplementary Table S2. The information of DUB library.

Gene	Species	DNA sequence	Plasmid vector	Predicted protein molecular weight
USP1	Homo sapiens	NM_003368.4	pCMV6Entry	88 kDa
USP2	Homo sapiens	NM_004205.3	pCMV7Entry	67.9 kDa
USP3	Homo sapiens	NM_006537.2	pCMV8Entry	58.7 kDa
USP4	Homo sapiens	NM_003363.2	pCMV9Entry	108.4 kDa
USP5	Homo sapiens	NM_001098536.1	pCMV10Entry	95.6 kDa
USP6	Homo sapiens	NM_004505.2	pCMV11Entry	158.5 kDa
USP7	Homo sapiens	NM_003470.1	pCMV12Entry	128.1 kDa
USP8	Homo sapiens	NM_005154.2	pCMV13Entry	127.3 kDa
USP10	Homo sapiens	NM_005153.2	pCMV14Entry	87 kDa
USP11	Homo sapiens	NM_004651.3	pCMV15Entry	109.6 kDa
USP12	Homo sapiens	NM_182488.1	pCMV16Entry	42.7 kDa
USP13	Homo sapiens	NM_003940.1	pCMV17Entry	97.1 kDa
USP14	Homo sapiens	NM_005151.3	pCMV18Entry	55.9 kDa
USP15	Homo sapiens	NM_006313.1	pCMV19Entry	109.1 kDa
USP16	Homo sapiens	NM_006447.2	pCMV20Entry	93.4 kDa
USP18	Homo sapiens	NM_017414.2	pCMV21Entry	42.8 kDa
USP19	Homo sapiens	NM_006677.1	pCMV22Entry	145.5 kDa
USP20	Homo sapiens	NM_006676.6	pCMV23Entry	101.8 kDa
USP21	Homo sapiens	NM_012475.4	pCMV24Entry	62.5 kDa
USP22	Homo sapiens	NM_015276.1	pCMV25Entry	59.8 kDa
USP24	Homo sapiens	NM_015306.2	pCMV26Entry	294.2 kDa
USP25	Homo sapiens	NM_013396.3	pCMV27Entry	122 kDa
USP26	Homo sapiens	NM_031907.1	pCMV28Entry	103.9 kDa
USP27X	Homo sapiens	NM_001145073.1	pCMV29Entry	49.5 kDa
USP29	Homo sapiens	NM_020903.2	pCMV30Entry	104 kDa
USP30	Homo sapiens	NM_032663.3	pCMV31Entry	58.3 kDa
USP33	Homo sapiens	NM_015017.3	pCMV32Entry	106.5 kDa
USP35	Homo sapiens	NM_020798.1	pCMV33Entry	113.2 kDa
USP36	Homo sapiens	NM_025090.3	pCMV34Entry	122.7 kDa
USP37	Homo sapiens	NM_020935.1	pCMV35Entry	110 kDa
USP38	Homo sapiens	NM_032557.4	pCMV36Entry	116.4 kDa
USP39	Homo sapiens	NM_006590.2	pCMV37Entry	65.2 kDa
USP40	Homo sapiens	NM_018218.1	pCMV38Entry	141.4 kDa
USP42	Homo sapiens	NM_032172.1	pCMV39Entry	144.2 kDa
USP43	Homo sapiens	NM_153210.3	pCMV40Entry	122.6 kDa
USP44	Homo sapiens	NM_032147.2	pCMV41Entry	81 kDa
USP45	Homo sapiens	NM_001080481.1	pCMV42Entry	91.6 kDa
USP46	Homo sapiens	NM_022832.2	pCMV43Entry	42.3 kDa

USP47	Homo sapiens	NM_017944.3	pCMV44Entry	147 kDa
USP48	Homo sapiens	NM_032236.4	pCMV45Entry	118.9 kDa
USP49	Homo sapiens	NM_018561.3	pCMV46Entry	73.3 kDa
USP50	Homo sapiens	NM_203494.1	pCMV47Entry	38.2 kDa
USP51	Homo sapiens	NM_201286.2	pCMV48Entry	79.6 kDa
USP52	Homo sapiens	NM_001127460.1	pCMV49Entry	135.2 kDa
USP53	Homo sapiens	NM_019050.2	pCMV50Entry	120.6 kDa

Supplementary Table S3. USPs-induced GFP intensity changes and correlation with OS in lung cancer patients.

Function	USPs	Changes of GFP intensity		KM Plotter in LC	
		(Mean±SD) (<1 in red)	*P* value (<0.001 in red)	(Negative correlation with OS in red)	logrank *P* value
NA	Ctl	1.000±0.009	NA	NA	NA
Pro-autophagy	EBSS	0.858±0.027	5.21E-05	NA	NA
	USP19	0.829±0.057	0.0030	Irrelevant	0.7359
	USP7	0.840±0.036	0.0002	Positive	2.50E-08
	**USP5**	**0.861±0.015**	**4.28E-07**	**Negative**	**5.80E-05**
	USP14	0.862±0.047	0.0057	Irrelevant	0.6604
	USP27X	0.869±0.049	0.0067	Positive	1.20E-13
	USP2	0.871±0.076	0.0580	Positive	1.90E-05
	USP35	0.872±0.112	0.1794	Irrelevant	0.7216
	USP50	0.874±0.054	0.0054	Not detected	Not detected
	USP12	0.883±0.030	0.0007	Positive	1.10E-05
	USP22	0.890±0.032	0.0012	Positive	3.40E-05
	USP51	0.890±0.035	0.0023	Positive	6.90E-14
	USP39	0.890±0.072	0.1984	Negative	0.0003
	USP18	0.892±0.016	6.28E-05	Irrelevant	0.5042
	USP3	0.892±0.034	0.0028	Positive	1.60E-10
	USP1	0.896±0.017	5.70E-05	Irrelevant	0.2600
	USP20	0.901±0.022	0.0004	Positive	8.70E-06
	USP46	0.909±0.020	0.0006	Irrelevant	0.068
	USP40	0.909±0.039	0.0079	Positive	6.20E-07
	USP38	0.916±0.048	0.0413	Positive	2.00E-08
	USP52	0.925±0.072	0.1407	Positive	0.0004
	USP10	0.948±0.005	0.0113	Negative	0.0006
	USP29	0.949±0.036	0.0841	Negative	4.30E-05
	USP13	0.962±0.050	0.3442	Positive	0.0014
	USP37	0.986±0.048	0.7363	Positive	5.50E-06
	USP4	0.993±0.035	0.8255	Irrelevant	0.0730
	USP25	0.993±0.058	0.8892	Positive	2.20E-09
Anti-autophagy	USP8	1.282±0.015	1.20E-10	Positive	1.40E-06
	USP21	1.210±0.101	0.0078	Positive	0.0101
	USP45	1.195±0.054	0.0003	Positive	0.0001
	USP6	1.162±0.031	3.34E-05	Irrelevant	0.5281
	USP53	1.141±0.091	0.0356	Positive	4.50E-14

	USP48	1.075±0.008	0.0004	Positive	4.60E-08
	USP49	1.066 ±0.043	0.0550	Positive	0.0072
	USP15	1.063±0.034	0.0617	Positive	6.40E-07
	USP36	1.059±0.018	0.0192	Positive	0.0008
	USP26	1.057±0.120	0.5660	Negative	0.002
	USP42	1.054±0.072	0.2770	Positive	0.0027
	USP47	1.047±0.030	0.0718	Positive	2.50E-09
	USP33	1.034±0.052	0.4418	Positive	0.0003
	USP24	1.034±0.008	0.0310	Positive	0.0007
	USP44	1.033±0.055	0.3962	Positive	0.0161
	USP16	1.032±0.034	0.2769	Positive	2.90E-06
	USP11	1.004±0.035	0.8606	Irrelevant	0.3357
	USP43	1.004±0.132	0.9650	Irrelevant	0.6645
	USP30	1.001±0.037	0.2384	Positive	0.0009

The GFP fluorescent intensity was normalized to vector control. Three independent experiments were performed. Data were presented as mean ± SD. Comparisons were performed with two-tailed Student's t test.
