# Supplementary material for: USP5-Beclin 1 axis overrides p53-dependent senescence and drives Kras-induced tumorigenicity
Source: Nat Commun. 2022 Dec 17;13:7799. doi: 10.1038/s41467-022-35557-y (PMC9759531; doi:10.1038/s41467-022-35557-y)
Supplement: Supplementary file 3 — Reporting Summary [file 41467_2022_35557_MOESM3_ESM.pdf]

nature portfolio

Corresponding author(s): Yang Wang and Zhi-Xiong Jim Xiao

Last updated by author(s): Dec 1, 2022

Reporting Summary

Nature Portfolio wishes to improve the reproducibility of the work that we publish. This form provides structure for consistency and transparency in reporting. For further information on Nature Portfolio policies, see our Editorial Policies and the Editorial Policy Checklist.

Statistics

For all statistical analyses, confirm that the following items are present in the figure legend, table legend, main text, or Methods section.

n/a	Confirmed	
☐	☒	The exact sample size (*n*) for each experimental group/condition, given as a discrete number and unit of measurement
☐	☒	A statement on whether measurements were taken from distinct samples or whether the same sample was measured repeatedly
☐	☒	The statistical test(s) used AND whether they are one- or two-sided<br>*Only common tests should be described solely by name; describe more complex techniques in the Methods section.*
☒	☐	A description of all covariates tested
☐	☒	A description of any assumptions or corrections, such as tests of normality and adjustment for multiple comparisons
☐	☒	A full description of the statistical parameters including central tendency (e.g. means) or other basic estimates (e.g. regression coefficient) AND variation (e.g. standard deviation) or associated estimates of uncertainty (e.g. confidence intervals)
☐	☒	For null hypothesis testing, the test statistic (e.g. *F*, *t*, *r*) with confidence intervals, effect sizes, degrees of freedom and *P* value noted<br>*Give P values as exact values whenever suitable.*
☒	☐	For Bayesian analysis, information on the choice of priors and Markov chain Monte Carlo settings
☒	☐	For hierarchical and complex designs, identification of the appropriate level for tests and full reporting of outcomes
☐	☒	Estimates of effect sizes (e.g. Cohen's *d*, Pearson's *r*), indicating how they were calculated

Our web collection on statistics for biologists contains articles on many of the points above.

Software and code

Policy information about availability of computer code

Data collection	NDP.scan SQ (v1.0)<br>Bio-Rad CFX Manager (v3.1)<br>LAS X (v 3.3.0.16779)<br>Image Lab (v 5.1)<br>NIS-Elements F (v 3.00, SP7(Build 547))<br>Countess II FL (v 1.0.238)<br>FACSDiva (v8.0.1)<br>CellQuestTM Pro (v6.0)
Data analysis	NDP.view (v 2.3)<br>QuPath (v 0.3.2)<br>GraphPad (v 8.3.0)<br>ImageJ (v 1.53k)<br>Adobe illustrator (v 25.2)<br>Microsoft Excel (v 2107)<br>SnapGene (v 4.3.6)<br>ZDOCK (v 3.02)<br>PyMOL (v 1.8)<br>Gromacs (v5.0.7)

FlowJo (v10.4.0)

For manuscripts utilizing custom algorithms or software that are central to the research but not yet described in published literature, software must be made available to editors and reviewers. We strongly encourage code deposition in a community repository (e.g. GitHub). See the Nature Portfolio guidelines for submitting code & software for further information.

Data

Policy information about availability of data

All manuscripts must include a data availability statement. This statement should provide the following information, where applicable:

- Accession codes, unique identifiers, or web links for publicly available datasets
- A description of any restrictions on data availability
- For clinical datasets or third party data, please ensure that the statement adheres to our policy

All relevant data supporting the findings of this study are available within the article.

Human research participants

Policy information about studies involving human research participants and Sex and Gender in Research.

Reporting on sex and gender	No human research participants
Population characteristics	No human research participants
Recruitment	No human research participants
Ethics oversight	No human research participants

Note that full information on the approval of the study protocol must also be provided in the manuscript.

Field-specific reporting

Please select the one below that is the best fit for your research. If you are not sure, read the appropriate sections before making your selection.

☒ Life sciences ☐ Behavioural & social sciences ☐ Ecological, evolutionary & environmental sciences

For a reference copy of the document with all sections, see nature.com/documents/nr-reporting-summary-flat.pdf

Life sciences study design

All studies must disclose on these points even when the disclosure is negative.

Sample size	Sample sizes were determined based on the means and variation of previous pilot or published experiments (Poillet-Perez et al, Nature, 2018; Manchado et al, Nature, 2016; Saliakoura et al, Nature Cell Biology,2020; Zhang et al, Nature Communications, 2017; Wang et al, Nature Communications, 2016).
Data exclusions	No data were excluded.
Replication	For in vitro experiments, at least three biologically independent experiments were performed for all experiments. For in vivo experiments, n=5 or 8 mice/group mice were used. All experiments were reliably reproduced.
Randomization	Six-week-old female BALB/C nude mice or KrasLSL-G12D/+ C57BL/6 mice were randomly divided into different experimental groups and housed under standard conditions.
Blinding	For animal experiments, animals of indicated groups were selected and assigned randomly, data collection and analysis were performed blindly. We performed other experiments, such as western blotting and colony formation assays, in a non-blinded manner, since the experimental design was complicated and the researchers were limited.

Reporting for specific materials, systems and methods

We require information from authors about some types of materials, experimental systems and methods used in many studies. Here, indicate whether each material, system or method listed is relevant to your study. If you are not sure if a list item applies to your research, read the appropriate section before selecting a response.

Materials & experimental systems

n/a	Involved in the study
☐	☒ Antibodies
☐	☒ Eukaryotic cell lines
☒	☐ Palaeontology and archaeology
☐	☒ Animals and other organisms
☒	☐ Clinical data
☒	☐ Dual use research of concern

Methods

n/a	Involved in the study
☒	☐ ChIP-seq
☐	☒ Flow cytometry
☒	☐ MRI-based neuroimaging

Antibodies

Antibodies used

For western blot analyses, antibodies specific for USP5 (sc-390943) and p53 (sc-126 or sc-6243) were purchased from Santa Cruz Biotechnology (CA, USA); antibodies for Beclin 1 (#3495 or #4122), LC3B (#2775), p62/SQSTM1 (#5114), PARP (#9532), Caspase-3 (#9662), HA (#2367), Flag (#14793) and Ras (#8955) were purchased from Cell Signaling Technology (Danvers, MA, USA); antibodies for p21 (CY5543), MDM2 (CY3612) and GAPDH (AB0037) were purchased from Abways (Shanghai, China); antibody specific for His (230001) was purchased from Zen BioScience (Chengdu, China).
For IHC analyses, antibodies for USP5 (ab244290), Beclin 1 (ab62557), LC3B (ab48394), and p62 (ab56416) were purchased from Abcam (Cambridge, MA, USA); antibody for PAI-1 (#11907) was purchased from Cell Signaling Technology; antibody for human p53 (sc-126) was purchased from Santa Cruz Biotechnology; antibody for mouse p53 (NCL-L-p53-CM5p) was purchased from Leica Biosystems (Newcastle, UK).
For IF analyses, antibodies for USP5 (sc-390943) or p53 (sc-126) were purchased from Santa Cruz Biotechnology; antibody for Beclin 1 was purchased from Abcam; antibody for HA (#2367) was from Cell Signaling Technology. Rhodamine (TRITC)-conjugated AffiniPure Donkey Anti-Rabbit IgG (#711-025-152) and Fluorescein (FITC)-conjugated AffiniPure Donkey Anti-mouse IgG (#715-095-150) were purchased from Jackson Immuno Research (PA, USA).

Validation

anti-USP5 mouse monoclonal, Santa Cruz Biotech cat.no. sc-390943, WB 1:200, IF 1:100.
https://www.scbt.com/zh/p/usp5-antibody-c-11
Validated by the company and following publication: Baoshan Cai, Jian Zhao, Yuling Zhang, et al. USP5 attenuates NLRP3 inflammasome activation by promoting autophagic degradation of NLRP3. Autophagy. 2021 Sep 5;1-15. doi: 10.1080/15548627.2021.1965426.

anti-p53 mouse monoclonal, Santa Cruz Biotech cat.no.sc-126, WB 1:200, IHC 1:200, IF:1:100.
https://www.scbt.com/p/p53-antibody-do-1
Validated by the company and the following publication: Liebl MC, Moehlenbrink J, Becker H, Raddatz G, et al. DAZAP2 acts as specifier of the p53 response to DNA damage. Nucleic Acids Res. 2021 Mar 18;49(5):2759-2776. doi: 10.1093/nar/gkab084.

anti-p53 rabbit polyclonal, Santa Cruz Biotech cat.no.sc-6243, WB 1:200.
https://www.scbt.com/p/p53-antibody-fl-393
Validated by the company and the following publication: Kwan Young Lee, Kathryn A Jewett, Hee Jung Chung, et al. Loss of fragile X protein FMRP impairs homeostatic synaptic downscaling through tumor suppressor p53 and ubiquitin E3 ligase Nedd4-2. Hum Mol Genet. 2018 Aug 15;27(16):2805-2816. doi: 10.1093/hmg/ddy189.

anti-Beclin 1 rabbit monoclonal, Cell Signaling cat. no. #3495, WB 1:1000.
https://www.cellsignal.cn/products/primary-antibodies/beclin-1-d40c5-rabbit-mab/3495
Validated by the company and the following publication: Chenglong Xie, Xu-Xu Zhuang, Zhangming Niu, et al. Nat Biomed Eng. 2022 Jan;6(1):76-93. doi: 10.1038/s41551-021-00819-5.

anti-Beclin 1 mouse monoclonal, Cell Signaling cat. no. #4122, WB 1:1000.
https://www.cellsignal.cn/products/primary-antibodies/beclin-1-2a4-mouse-mab/4122
Validated by the company and the following publication: Lin Lyu, Zheng Chen, Nami McCarty. Autophagy. 2022 Apr;18(4):783-798. doi: 10.1080/15548627.2021.1956105.

anti-LC3B rabbit polyclonal, Cell Signaling cat. no. #2775, WB 1:1000.
https://www.cellsignal.cn/products/primary-antibodies/lc3b-antibody/2775
Duc-Vinh Pham, Pil-Hoon Park. Adiponectin triggers breast cancer cell death via fatty acid metabolic reprogramming. J Exp Clin Cancer Res. 2022 Jan 5;41(1):9. doi: 10.1186/s13046-021-02223-y.

anti-p62 rabbit polyclonal, Cell Signaling cat. no. #5114, WB 1:1000.
https://www.cellsignal.cn/products/primary-antibodies/sqstm1-p62-antibody/5114
Validated by the company and following publication: Limei Wu, Srinivas Chatla, Qiqi Lin, et al. Quinacrine-CASIN combination overcomes chemoresistance in human acute lymphoid leukemia. Nat Commun. 2021 Nov 26;12(1):6936. doi: 10.1038/s41467-021-27300-w.

anti-PARP rabbit monoclonal, Cell Signaling cat. no.#9532, WB 1:1000.
https://www.cellsignal.com/products/primary-antibodies/parp-46d11-rabbit-mab/9532
Validated by the company and following publication: Yi Du, Hirohito Yamaguchi, Yongkun Wei, et al. Blocking c-Met–mediated PARP1 phosphorylation enhances anti-tumor effects of PARP inhibitors. Nat Med. 2016 22, pages194–201. doi: 10.1038/nm.4032

anti-Caspase-3 rabbit Polyclonal, Cell Signaling cat. no.#9662, WB 1:1000
https://www.cellsignal.com/products/primary-antibodies/caspase-3-antibody/9662
Validated by the company and following publication: Yupeng Wang, Wenqing Gao, Xuyan Shi, et al. Chemotherapy drugs induce pyroptosis through caspase-3 cleavage of a gasdermin. Nature. 2017 volume 547, pages99–103. doi: 10.1038/nature22393.

anti-HA mouse monoclonal, Cell Signaling cat. no. #2367, WB 1:1000, IF 1:400.
https://www.cellsignal.cn/products/primary-antibodies/ha-tag-6e2-mouse-mab/2367
Validated by the company and following publication: Xuxu Gou, Meenakshi Anurag, Jonathan T Lei, et al. Transcriptional Reprogramming Differentiates Active from Inactive ESR1 Fusions in Endocrine Therapy-Refractory Metastatic Breast Cancer. Cancer Res. 2021 Dec 15;81(24):6259-6272. doi: 10.1158/0008-5472.CAN-21-1256.

anti-Flag rabbit monoclonal, Cell Signaling cat. no. #14793, WB 1:1000.
https://www.cellsignal.cn/products/primary-antibodies/dykddddk-tag-d6w5b-rabbit-mab-binds-to-same-epitope-as-sigma-s-anti-flag-m2-antibody/14793
Validated by the company and following publication: Minghuan Mao, Liang Yang, Jingyao Hu, et al. Oncogenic E3 ubiquitin ligase NEDD4 binds to KLF8 and regulates the microRNA-132/NRF2 axis in bladder cancer. Exp Mol Med. 2022 Jan;54(1):47-60. doi: 10.1038/s12276-021-00663-2.

anti-Ras rabbit monoclonal, Cell Signaling cat. no. #8955, WB 1:1000.
https://www.cellsignal.cn/products/primary-antibodies/ras-d2c1-rabbit-mab/8955
Validated by the company and following publication: Yusuke Inoue, Ana Nikolic, Dylan Farnsworth, et al. Extracellular signal-regulated kinase mediates chromatin rewiring and lineage transformation in lung cancer. Elife. 2021 Jun 14;10:e66524. doi: 10.7554/eLife.66524.

anti-p21 rabbit monoclonal, rabbit monoclonal, abways cat. no. CY5543, WB 1:1000.
http://www.abways.cn/showproduct.asp?cid=CY5543
Validated by the company and following publication: Xin Wang, Qing Zhang, Changji Li, et al. Fibulin-3 Has Anti-Tumorigenic Activities in Cutaneous Squamous Cell Carcinoma. J Invest Dermatol. 2019 Aug;139(8):1798-1808.e5. doi: 10.1016/j.jid.2019.01.022.

anti-GAPHD rabbit monoclonal, abways cat. no. AB0037, WB 1:5000.
http://www.abways.cn/showproduct.asp?cid=AB0037
Validated by the company and following publication: Jianhua Mao, Lie Ma, Yan Shen, et al. Arsenic circumvents the gefitinib resistance by binding to P62 and mediating autophagic degradation of EGFR in non-small cell lung cancer. Cell Death Dis. 2018 Sep 20;9(10):963. doi: 10.1038/s41419-018-0998-7.

anti-His mouse monoclonal, Zen Bio cat. no. 230001, WB 1:1000.
http://www.zen-bio.cn/prod_view.aspx?IsActiveTarget=True&TypeId=160&Id=454563&FId=t3:160:3
Validated by the company and following publication: Yanqun Zhao, Qiang Meng, Yujie Lai, et al. Structural and mechanistic insights into polymyxin resistance mediated by EptC originating from Escherichia coli. FEBS J. 2019 Feb;286(4):750-764. doi: 10.1111/febs.14719.

anti-USP5 Rabbit polyclonal, Abcam cat. no. ab244290, IHC 1:400
https://www.abcam.cn/usp5-antibody-ab244290.html
Validated by the company and following publication: Jiajia Li, Haiying Li, Weijian Zhu, et al. Deubiquitinase inhibitor degrasyn suppresses metastasis by targeting USP5-WT1-E-cadherin signalling pathway in pancreatic ductal adenocarcinoma. J Cell Mol Med. 24:1370-1382 (2020). doi: 10.1111/jcmm.14813

anti-Beclin1 Rabbit polyclonal, Abcam cat. no. ab62557, IHC 1:400
https://www.abcam.cn/beclin-1-antibody-ab62557.html
Validated by the company and following publication: Shiwen Lu, Lifei Yu, Hao Liu. Trimetazidine alleviates hypoxia/reoxygenation-induced apoptosis in neonatal mice cardiomyocytes via up-regulating HMGB1 expression to promote autophagy. J Recept Signal Transduct Res. 2021 Apr;41(2):170-179. doi: 10.1080/10799893.2020.1800736.

anti-LC3B Rabbit polyclonal, Abcam cat.no.ab48394, IHC 1:400
https://www.abcam.cn/lc3b-antibody-autophagosome-marker-ab48394.html
Validated by the company and following publication: Yabing Chen, Jing Wang, Dihui Xu, et al. m6A mRNA methylation regulates testosterone synthesis through modulating autophagy in Leydig cells. Autophagy. 2021 Feb;17(2):457-475. doi: 10.1080/15548627.2020.1720431.

anti-p62 Mouse monoclonal, Abcam cat. no. ab56416, IHC 1:400
https://www.abcam.cn/sqstm1--p62-antibody-2c11-bsa-and-azide-free-ab56416.html
Validated by the company and following publication: Christine Zimmermann, Nadine Krämer, Steffi Krauter, et al. Autophagy interferes with human cytomegalovirus genome replication, morphogenesis, and progeny release. Autophagy. 2021 Mar;17(3):779-795. doi: 10.1080/15548627.2020.1732686.

anti-PAI-1 Rabbit monoclonal, Cell Signaling cat. no.11907, IHC 1:800
https://www.cellsignal.cn/products/primary-antibodies/pai-1-d9c4-rabbit-mab/11907
Validated by the company and following publication: Subir Kumar Juin, Sathnur Pushpakumar, Utpal Sen. GYY4137 Regulates Extracellular Matrix Turnover in the Diabetic Kidney by Modulating Retinoid X Receptor Signaling. Biomolecules. Biomolecules. 2021

Oct 7;11(10):1477. doi: 10.3390/biom11101477.

anti-p53 Rabbit polyclonal, Leica Biosystems cat. no. P53-CM5P-L, IHC 1:400.
https://shop.leicabiosystems.com/en-cn/pid-P53-CM5P-L

TRITC AffiniPure donkey polyclonal anti-rabbit IgG (Jackson Immuno Research cat.no. 711-025-152), IF 1:160
https://www.jacksonimmuno.com/catalog/products/711-025-152
Validated by the company and the following publication: Azarm K, Bhardwaj A, Kim E, et al. Persistent telomere cohesion protects aged cells from premature senescence. Nat Commun. 2020 Jul 3;11(1):3321. doi: 10.1038/s41467-020-17133-4.

FITC AffiniPure donkey polyclonal anti-mouse IgG (Jackson Immuno Research cat.no. 715-095-151), IF 1:160
https://www.jacksonimmuno.com/catalog/products/715-095-150
Validated by the company and the following publication: Huang G, Ge G, Izzi V, et al. α3 Chains of type V collagen regulate breast tumour growth via glypican-1. Nat Commun. 2017 Jan 19;8:14351. doi: 10.1038/ncomms14351.

Eukaryotic cell lines

Policy information about cell lines and Sex and Gender in Research

Cell line source(s): NCI-H292 (CRL-1848) was obtained from ATCC (Manassas, VA, USA). A549 (BNCC337696) was obtained from BeNa Culture Collection (Beijing, China). SK-LU-1 (SNL-492) and NCI-H358 (SNL-392) were obtained from Sunncell Biotech (Wuhan, China). NCI-H1299 (CL-0165) and H1975 (CL-0298) cells were obtained from Procell Life Science&Technology (Wuhan, China). HEK293FT (R70007) cells were from Thermo Fisher Scientific (Waltham, MA, USA).

Authentication: All cell lines were authenticated by morphology check and growth curve analysis.

Mycoplasma contamination: Cell lines were routinely tested and found negative for mycoplasma infection by the vender.

Commonly misidentified lines (See ICLAC register): None of the cell lines used in this study is found in the database of commonly misidentified cell lines.

Animals and other research organisms

Policy information about studies involving animals; ARRIVE guidelines recommended for reporting animal research, and Sex and Gender in Research

Laboratory animals: The Becn1 conditional knockout mice (Becn1flox/flox) were obtained from Shanghai Model Organisms Center (Shanghai, China). KrasLSL-G12D/+ mice are provided by Dr. Chong Chen (Sichuan University, China). Female BALB/C nude mice were obtained from Model Animal Research Center of Nanjing University (Nanjing, China).

Wild animals: No wild animals were used in this study.

Reporting on sex: For in vivo tumor progression, only female nude mice were applied. For genetic mouse models, both male and female mice were applied.

Field-collected samples: This study did not involve field-collected samples.

Ethics oversight: All animal experiments in this study were approved by the Institutional Animal Care and Use Committee (IACUC) of Sichuan University (No.20210308015), and the procedures were performed according to the guidelines established by the China Council on Animal Care.

Note that full information on the approval of the study protocol must also be provided in the manuscript.

Flow Cytometry

Plots

Confirm that:

- [x] The axis labels state the marker and fluorochrome used (e.g. CD4-FITC).
- [x] The axis scales are clearly visible. Include numbers along axes only for bottom left plot of group (a 'group' is an analysis of identical markers).
- [x] All plots are contour plots with outliers or pseudocolor plots.
- [x] A numerical value for number of cells or percentage (with statistics) is provided.

Methodology

Sample preparation: Please see Materials and Methods, in screening for DUB regulation of autophagy, the 293FT cells were infected with the recombinant lentivirus expressing pLenti-CMV-RFP-GFP-LC3 and selected for stable cells displaying uniform and evenly distributed red/green fluorescence by flow cytometer (FACS AriaIII, BD). These 293FT-RFP-GFP-LC3 cells were transfected with USP genes, and then subjected to FACS analyses (FACS CaliburTM, BD Biosciences) for the changes of GFP fluorescence

	intensity.<br>In addition, ROS level was deterrmined by using DCFH-DA (Beyotime, S0033). Briefly, cells was trypsinized, washed with PBS and incubated with 10μM DCFH-DA at 37°C for 30 min to load the fluorescent dye. Afterward, cells were washed with PBS and subjected to ROS detection by FACS.
Instrument	The data were acquired using FACS AriaIII (BD Biosciences) and FACS CaliburTM (BD Biosciences).
Software	FACSDiva (BD Biosciences) is used to sort cells.<br>BD CellQuestTM Pro (BD Biosciences) is used to collect data.<br>FlowJo Software is used to analyze cytometry data.
Cell population abundance	500,000 293FT-RFP-GFP-LC3 cells were gated and sorted for uniform and evenly distributed red/green fluorescence cells.
Gating strategy	Based on forward and side scatter (FSC/SSC plot) properties a gate was set on the live cell population; dead cells were excluded, and then were compared GFP or ROS level.

☒ Tick this box to confirm that a figure exemplifying the gating strategy is provided in the Supplementary Information.
